# Tick salivary cystatin Iristatin limits the virus replication in skin of tick-borne encephalitis virus–infected mice

**DOI:** 10.1007/s00436-024-08441-5

**Published:** 2025-01-17

**Authors:** Helena Langhansová, Zuzana Beránková, Ritesh Khanna, Jan Kotál, Michail Kotsyfakis, Martin Palus, Jaroslava Lieskovská

**Affiliations:** 1 Department of Medical Biology, Faculty of Science, University of South Bohemia, Branišovská 1760, CZ-37005 České Budějovice, Czech Republic; 2 Institute of Parasitology, Biology Centre of Czech Academy of Sciences, Branišovská 1160/31, CZ-37005 České Budějovice, Czech Republic; 3 Institute of Molecular Biology and Biotechnology, Foundation for Research and Technology-Hellas, N. Plastira 100, 70013 Heraklion, Crete Greece; 4 Department of Virology, Veterinary Research Institute, Hudcova 70, CZ-62100 Brno, Czech Republic

**Keywords:** Cystatin, Flavivirus, Tick, Tick-borne encephalitis virus, Virus replication

## Abstract

Tick-borne encephalitis virus (TBEV) is flavivirus transmitted to the host via tick saliva which contains various molecules with biological impacts. One of such molecules is Iristatin, a cysteine protease inhibitor from *Ixodes ricinus* that has been shown to have immunomodulatory properties. To characterize Iristatin in the relation to TBEV, we investigate whether this tick inhibitor has any capacity to influence TBEV infection. Mice were intradermally infected by TBEV with or without Iristatin and the viral multiplication was determined in skin and brain tissues by RT-PCR two and 5 days after infection. The viral RNA was detected in both intervals in skin and increased by time. The application of Iristatin caused a reduction in viral RNA in skin but not in the brain of infected mice 5 days post-infection. Moreover, anti-viral effect of Iristatin on skin was accompanied by a significant decline of interferon-stimulated gene 15 gene expression. The effect of Iristatin on TBEV replication was tested also in vitro in primary macrophages and dendritic cells; however, no changes were observed suggesting no direct interference of Iristatin with virus replication. Still, the Iristatin caused a suppression of Erk1/2 phosphorylation in TBEV-infected dendritic cells and had the anti-apoptotic effect. This is the first report showing that a tick cystatin decreases the viral RNA in the host skin, likely indirectly through creating skin environment that is less supportive for TBEV replication. Assuming, that viral RNA reflects the amount of infectious virus, decline of TBEV in host skin could influence the tick biology or virus transmission during cofeeding.

**Supplementary Information:**

The online version contains supplementary material available at 10.1007/s00436-024-08441-5.

## Background

Tick-borne encephalitis virus (TBEV; *Orthoflavivirus encephalitidis*) is a positive single-stranded enveloped RNA flavivirus belonging to the *Flaviviridae* family. TBEV is endemic to Europe and northern Asia causing infection of the central nervous system. It is transmitted by ticks of the Ixodidae family, by *Ixodes ricinus* in Europe and by *Ixodes persulcatus* in Asia (Kazimirova et al. 2017). The virus first replicates in skin cells, including fibroblasts and keratinocytes, and in migratory monocytes and Langerhans dendritic cells (Hermance et al. 2016; Labuda et al. 1996). The latter are responsible for transporting the virus to the nearest lymphatic nodes followed by further spread to other lymphatic and non-lymphatic organs due to viremia (Ruzek et al. 2019). Afterwards, the target tissue (brain) is reached causing typical neurological pathology involving both direct virus-induced cellular damage and immune response-mediated pathology. Viral RNA replication occurs in replicative vesicles near the membrane of the endoplasmic reticulum. Virions enter the cell by clathrin-dependent endocytosis and mature virions depart via exocytosis (Ruzek et al. 2019).

Virus RNA molecules are sensed by Toll-like receptors (TLRs), RIG-I-like receptors (RIG-I and MDA5), and RNA-dependent protein kinases (PKR), inducing the production of inflammatory mediators and interferons (Kawai and Akira 2010). Interferon action is the most potent anti-viral reaction which is mediated through numerous IFN-inducible genes, e.g., ISG15, CXCL-10, OASL2, and IFIT2. Interferon production and signaling is essential for controlling TBEV infection in the periphery as well as in the brain (Weber et al. 2014).

TBEV is transmitted to the vertebrate host via tick saliva that is periodically released by the tick into a feeding cavity in the host’s skin (Nuttall 2019). Tick saliva contains a mixture of bioactive molecules that support the tick-feeding process and have anti-hemostatic and immunomodulatory effects (Kotal et al. 2015; Simo et al. 2017). It has been shown that tick-borne viruses, similarly to other tick-borne pathogens, exploit the immunosuppression evoked in the host by the tick (Kazimirova et al. 2017). The increase in virus acquisition resulting from the presence of tick saliva has been demonstrated for the Thogoto virus and later for TBEV (Jones et al. 1989; Labuda et al. 1993). The enhancing effect of salivary gland extract (SGE) on virus infection has been demonstrated for Powassan virus (POWV), a flavivirus closely related to TBEV, by showing that the amount of virus required to produce lethal animal infection was significantly lower when tick SGE was present (Hermance and Thangamani 2015). The tick salivary compounds responsible for these effects are yet to be revealed and are called SAT (saliva-assisted transmission) factors (Nuttall 2019). Today, no *I. ricinus* cystatin has been recognized to play any role in TBEV replication or infection. In the case of the mosquito-borne flaviviruses, including the dengue virus (DENV), West Nile virus (WNV), and Zika virus (ZIKV), several salivary molecules supporting or inhibiting the virus replication were identified (Conway et al. 2014; Oliveira et al. 2020b, a; Sun et al. 2020). The characterization of tick salivary proteins and their relation to tick-borne pathogen transmission is fuelled by a need to identify SAT factors with the potential to become a component of an anti-tick vaccine cocktail.

Iristatin is a tick salivary cystatin from *I. ricinus* (Kotal et al. 2019). Cystatins are a family of cysteine peptidase inhibitors found in several organisms and have been associated in ticks with blood acquisition, blood digestion, the modulation of host immune response, and tick biology (Chmelar et al. 2017). Cysteine proteases that are inhibited by cystatins include cathepsins B, H, L, C, and S, and are involved in the control of various cellular processes (Turk and Bode 1991). Iristatin specifically inhibits cathepsin C and L as evidenced by both its structure and function. In addition, Iristatin is a potent immunomodulator of the host immune system which has been demonstrated both in vivo and in vitro (Kotal et al. 2019). Iristatin attenuates Th1 and Th2 vertebrate host immune responses and inhibit ovalbumin-induced CD4 T cell proliferation and leukocyte recruitment. It also affects nitric oxide secretion and cytokines production from activated macrophages (Kotal et al. 2019). Recently, it has been reported that Iristatin suppresses innate immunity-dependent mannan-induced psoriasis-like inflammation through inhibition of IL-6/IL-23/IL17 axis cytokines (Wu et al. 2024). Due to these features, we hypothesized that Iristatin could influence the TBEV infection.

Herein we investigated the effect of Iristatin on TBEV infection in vivo and in vitro*.*

## Materials and methods

### Cells and virus

Bone marrow cells were obtained from the femurs and tibias of C57BL/6N mice by flushing the bones with RPMI 1640 medium. To obtain myeloid dendritic cells (DC), bone marrow cells were seeded at a concentration of 2 × 10^5^/ml in 10 cm diameter Petri dishes in a complete RPMI medium supplemented with 20 ng/ml recombinant mouse GM-CSF (Peprotech). The cells were cultured for 8 days at 37 °C and 5% CO_2_. On day three, 10 ml of fresh medium containing 20 ng/ml of GM-CSF was added and on day six, half of the volume (10 ml) was replaced with the fresh medium. On day eight, non-adherent cells were harvested and used as DC. For the derivation of bone-marrow macrophages (BMM), bone marrow cells were seeded at a concentration of 3 × 10^5^/ml in 10 cm diameter Petri dishes and cultured in complete RPMI medium supplemented with 30% of LCCM (L929 cell conditioned medium) for 7 days at 37 °C and 5% CO_2_. On day three, 10 ml of fresh medium (RPMI with 30% LCCM) was added. On day seven, adherent cells were harvested and used as BMM.

Hypr, a virulent strain of TBEV (the European subtype), was propagated in Vero E6 cells. Following virus infection, Vero E6 cells were incubated for 3 days, and when signs of cytopathic effect were visible, infectious medium was collected, and the virus titer was determined by plaque assay. As a control, the conditioned medium from non-infected Vero E6 cells was used.

Iristatin was prepared and used in an LPS-free recombinant form as previously described (Kotal et al. 2019).

### Mouse infection and the effect of Iristatin in vivo

Specific pathogen-free 7-week-old female mice of the C57BL/6N strain (10 mice per group) were intradermally inoculated with 10^2^ pfu of the Hypr TBEV strain in PBS into the lower back region, with or without 50 µg of recombinant Iristatin (in 50 µl volume). On days two and five post-infection, the mice were sacrificed by cervical dislocation and skin from the site of inoculation and the brains were dissected. Corresponding tissues from non-infected mice were used as control for gene expression analyses. Total RNA from the tissues was extracted using the NucleoSpin® RNA Kit (Macherey–Nagel). The RNA was reverse-transcribed and a 98 bp fragment of the TBEV NS1 protein region was amplified using the KAPA PROBE FAST Universal One-Step qRT-PCR Master Mix (2x) Kit (Kapa Biosystems), forward primer TGGAYTTYAGACAGAAYCAACACA, reverse primer TCCAGAGACTYTGRTCDGTGTGA and hydrolysis probe FAM-CCCATCACTCCWGTGTCAC-MGB-NFQ (Achazi et al. 2011). To detect *CXCL-10* and *CD115*, the primers/probe sets (Mm00445235_m1 and Mm01266652_m1) from Applied Biosystems were used. RT-qPCR analysis of *ISG15, TCRγ*, *OASL2*, and *IFIT2* was done using the KAPA SYBR FAST UNIVERSAL One-Step qRT-PCR Kit (Kapa Biosystems) and specific primers (*ISG15*: forward primer CAGTGATGCTAGTGGTACAG and reverse primer GCGTCAGAAAGACCTCATAG-3; *TCRγ*: forward primer TCCATAAGACTGGGACATACCT and reverse primer CCTGGGAGTCCAGGATAGTATT; *OASL2*: forward primer CCGTTCCCCGACCTGTATG and reverse primer CCTTCACCACCTTAATCACCCT; *IFIT2*: forward primer AGAACCAAAACGAGAGAGTGAAG and reverse primer TCCAGACGGTAGTTCGCAATG) according to the manufacturer’s protocol. Data were analyzed using Livak’s method (Livak and Schmittgen 2001) and normalized to the β-actin reference gene (*Actb* primers and probe Mm00607939 from Applied Biosystems in the case of TBEV, CXCL-10, and CD115 analysis and *Actb* primers sequence: forward CTCTGGCTCCTAGCACCATGAAGA and reverse GTAAAACGCAGCTCAGTAACAGTCCG in the case of ISG15, TCRγ, OASL2, and IFIT2 analysis).

### Hypr infection of primary macrophages and dendritic cells

BMM or DC were infected with the Hypr TBEV strain (multiplicity of infection (MOI) of 5). After virus adsorption for 1 h at 37 °C and 5% CO_2_, the cells were washed with medium and cultured in the presence or absence of Iristatin (6 μM) for 24, 48, and 72 h at 37 °C in 5% CO_2_. Afterwards, the culture medium and cells were collected. The culture medium was used for the quantification of the virus by plaque assay. Cells were used for the isolation of RNA to determine copies of the viral genome and *ISG15* and *CXCL-10* gene expression as described above.

### Quantification of TBE virus by plaque assay

Plaque assay was performed using A549 cells. Ten times serial dilutions of the samples were placed in 24-well plates and the suspension of A549 cells (concentration 5 × 10^5^ cells/ml) was added (300 μl per well). After adhesion (4 h later), the cells were overlaid with carboxymethylcellulose (1.5% in DMEM medium) and incubated for 5 days at 37 °C and 5% CO_2_. Afterwards, the plates were washed in 0.9% NaCl solution and the cells were stained with 0.1% naphthalene black in 6% acetic acid solution for 45 min. Virus-induced plaques were counted and virus titer was expressed as plaque forming units (pfu) per ml.

### PathScan intracellular signaling array

DC derived from bone marrow were seeded into a 24-well plate at the concentration of 1 × 10^6^ cells per ml and the next day activated by imiquimod at a final concentration of 2 µg/ml for 3 h in the presence or absence of Iristatin (3 µM). After 3 h, the cell protein lysates were prepared and analyzed using the PathScan® intracellular signaling array kit (#7323, Cell Signaling Technology) according to the manufacturer’s instruction. Chemiluminescence was measured in Aliance 4.7 Uvitec followed by analysis with UVIband software.

### Immunoblotting analysis

DC were seeded into a 24-well plate at a concentration of 1 × 10^6^ cells per ml. The next day, cells were infected by Hypr at MOI 5 with or without Iristatin. Cells were collected 1, 2, and 3 h after infection, lysed by RIPA buffer supplemented by protease inhibitors (EZBlock™ Protease Inhibitor Cocktail, EDTA-Free, BioVision), and analyzed on 8% SDS-PAGE. Upon transfer to nylon membrane, membranes were incubated with antibodies specific for phospho-Akt (Ser^473^) and phospho-Erk1/2 (p44/42 MAPK) (Thr^202^/Tyr^204^) and after membrane striping with antibodies against total Akt and Erk1/2 (Cell Signaling Technology) proteins. Proteins were visualized by ECL using WesternBright™ Quantum (Advansta) and intensities of bands were determined by ImageJ software. The relative phosphorylation was calculated as ratio of phosphorylated and non-phosphorylated (total) form of tested kinases. Experiment was performed three times.

### Measurement of active caspase-3 by flow cytometry

DC were let to adhere on 96-well plate at the concentration of 1 × 10^6^ cells per ml. Cells were infected or not by Hypr at MOI 5 in the presence or absence of Iristatin (6 µM) and after 24 h and 48 h cells were collected, washed once in PBS with 1% FBS, fixed, and stained with anti-caspase-3 antibody according to the protocol (FITC active caspase-3 apoptotic kit, BD Biosciences). Flow cytometry was performed on FACS Canto II flow cytometer and data were analyzed using FACS Diva software, v. 5.0 (BD Biosciences). Percentage of active caspase-3 positive cells was determined by number of positive cells in FITC channel.

### Statistical analysis

**Fig. 1:**
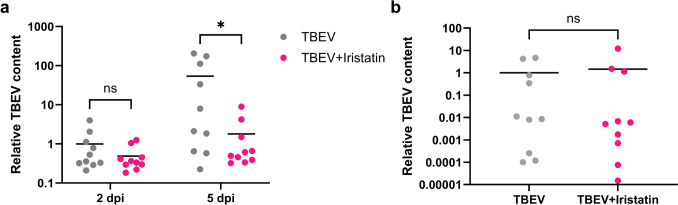
The effect of Iristatin on virus multiplication in mice. Ten mice per group were i.d. infected with Hypr ± Iristatin and viral genome RNA loads on indicated days post-infection were determined by RT-qPCR in the skin from inoculation site (**a**) and in the brain (**b**). mRNA expression was normalized to the Actb mRNA level. *p ≤ 0.05; ns = not significant

**Fig. 2:**
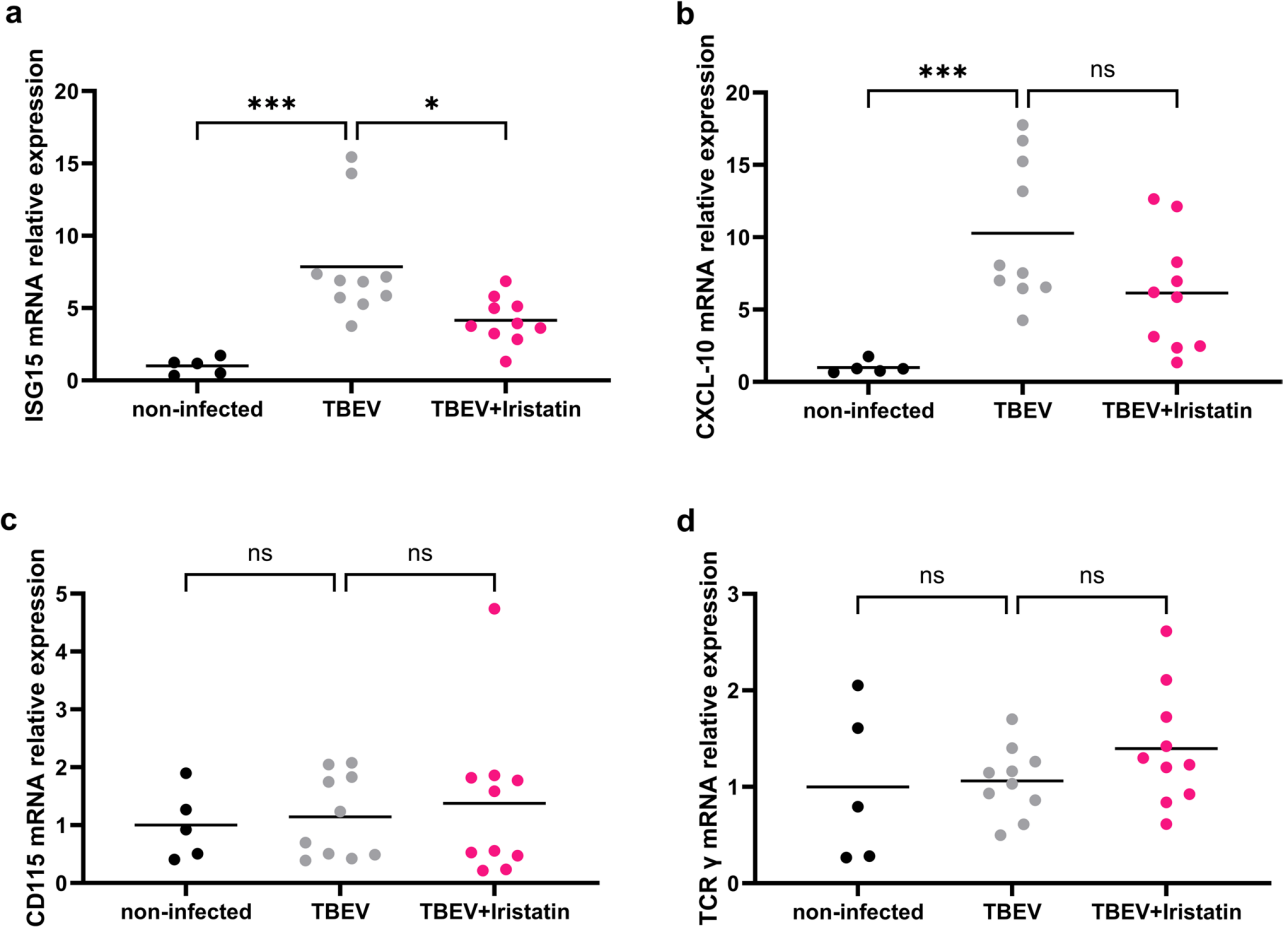
Iristatin affects the gene expression of interferon-responsive genes in the skin of TBEV-infected mice. Mice were i.d. infected with Hypr ± Iristatin and the gene expressions of ISG15 (**a**), CXCL-10 (**b**), CD115 (**c**), and TCRγ (**d**) were evaluated on day 5 in the skin of infected mice. mRNA expression was normalized to the Actb mRNA level and non-infected mice. *p ≤ 0.05; ***p ≤ 0.001; ns = not significant

**Fig. 3:**
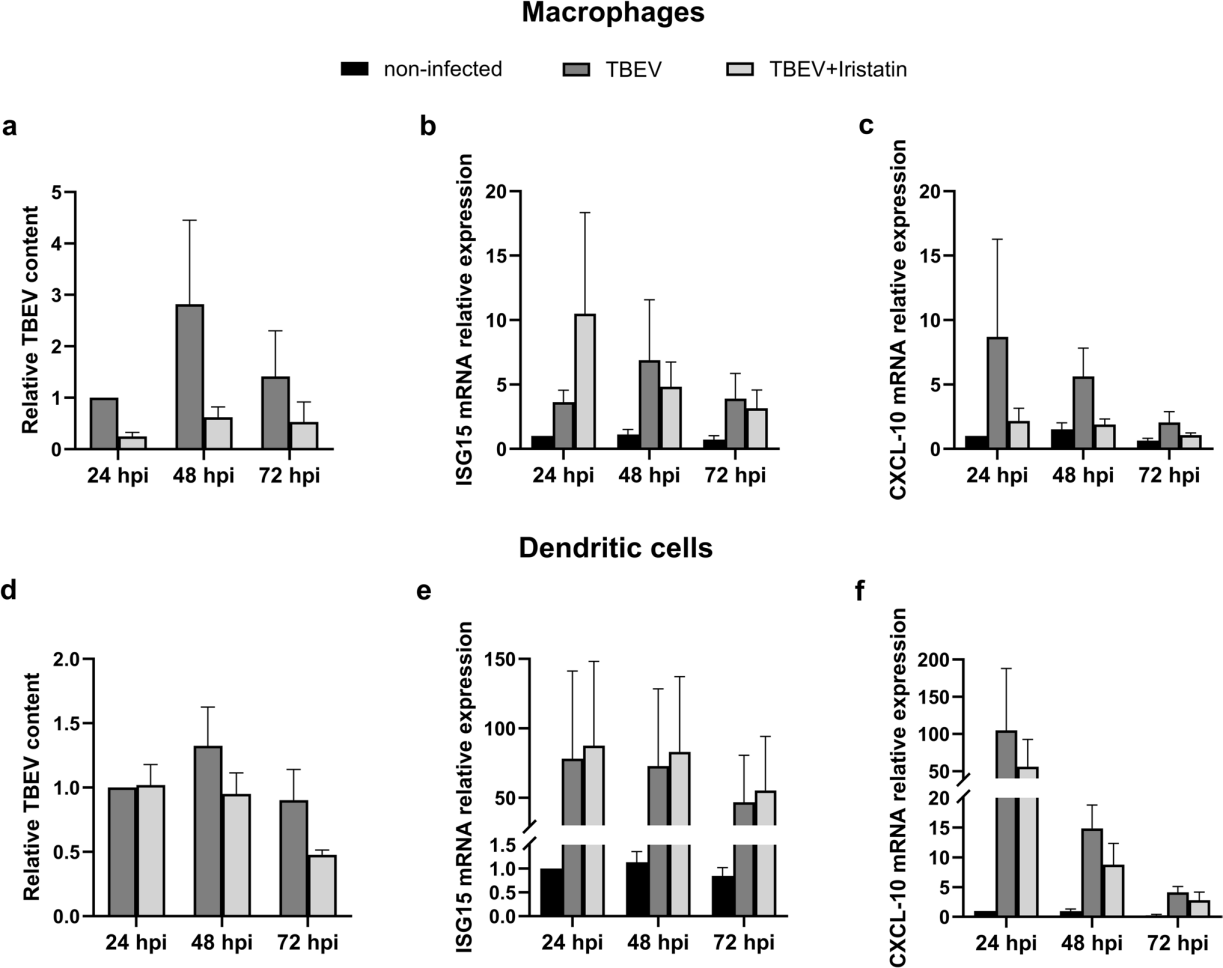
The effect of Iristatin on virus multiplication and ISG gene expression in primary bone marrow macrophages (BMM; **a, b, c**) and dendritic cells (DC; **d, e, f**). BMM and DC were infected with Hypr (MOI 5) and incubated for 24, 48, and 72 h in the presence or absence of Iristatin (6 μM). Viral genome RNA loads (**a, d**) were determined by RT-qPCR at indicated hours post-infection. mRNA expression of ISG15 (**b, e**) and CXCL-10 (**c, f**) were normalized to the Actb mRNA level and non-infected control. The mean of three independent experiments (+ SEM) is shown in all graphs. Differences between groups were not statistically significant

**Fig. 4:**
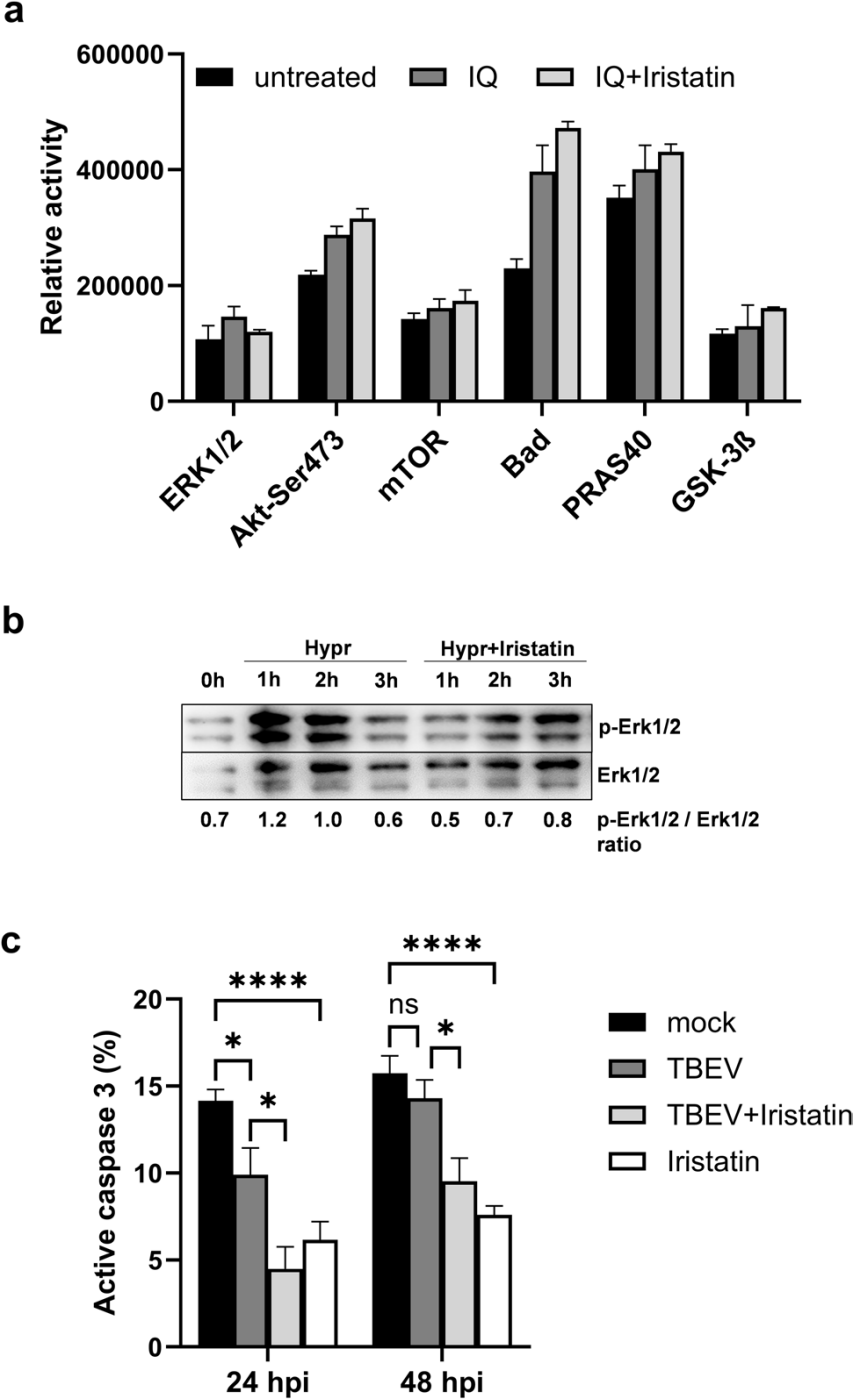
Iristatin interferes with signaling pathways activation and exerts anti-apoptotic effect in TBEV-infected dendritic cells. DC were activated by imiquimod (IQ; 2 µg/ml) for 3 h in the presence or absence of Iristatin (3 µM) and protein cell lysates were analyzed for the activation of signaling pathways using PathScan intracellular signaling array (**a**). DC were infected by Hypr at MOI 5 for indicated times in the presence or absence of Iristatin (3 µM) and then Erk1/2 phosphorylation was analyzed by immunoblotting. Membranes were re-probed to determine the level of total Erk1/2 proteins. Proteins were visualized by chemiluminescence and representative blot with relative phosphorylation is shown (**b**). DC were non-infected or infected by Hypr at MOI 5 in the presence or absence of Iristatin (6 µM) and the percentage of active caspase-3 positive cells was measured by flow cytometry (**c**). *p ≤ 0.05; ****p ≤ 0.0001 ns = not significant

Data from in vivo experiments are plotted as individual values and means. In vitro data are plotted as column averages plus standard error of mean. Figures were prepared in GraphPad Prism, version 10.3.0. Statistical analysis of Fig. 1a, Fig. 3, Fig. 4c, and Online Resource 2 was made using two-way analysis of variance (ANOVA) with either Šídák’s (Figs. 1a, 3a, d, Online Resource 2), Dunnett’s (Fig. 3b, c, e, f), or Tukey’s (Fig. 4c) multiple comparisons test. Figure 1b was analyzed using an unpaired *t*-test. Figure 2 and Online Resource 1 were analyzed by ordinary one-way ANOVA followed by Šídák’s post hoc test. The *p* values ≤ 0.05 were considered significant.

## Results

### Iristatin decreases the viral multiplication in the skin of TBEV-infected mice

First, to find out whether Iristatin has any ability to affect TBEV infection, we performed the following in vivo experiment. Mice were intradermally inoculated with the Hypr strain of TBEV, with or without Iristatin. On days two and five after infection, the viral loads were determined for skin (site of inoculation) and brain matter. The viral RNA was detected in the skin already 2 days upon infection and significantly increased in time (*p* = 0.016) in TBEV-infected mice. In the group, where Iristatin was administered with the virus, a decreased amount of viral RNA was found in the skin when compared to control TBEV-infected mice; the difference reached a statistical significance on day five post-infection (*p* = 0.0122) (Fig. 1a). In the brain, the viral RNA was detected 5 dpi; however, the amount was not affected by Iristatin treatment (Fig. 1b). Results show that Iristatin has a capacity to restrict virus load in skin with no apparent consequences to further dissemination within the host under used experimental conditions.

### Gene induction of interferon-stimulated genes is negatively affected by Iristatin in skin of TBEV-infected mice

Virus infection induces interferons (IFN) and consequently IFN-stimulated genes (ISGs). To find out whether a decrease of virus load observed in the presence of Iristatin results in a decrease of IFN or ISGs, we measured gene expression of IFN-β and four interferon-stimulated genes, namely ISG15, CXCL-10, OASL2, and IFIT2 in skin tissue two and 5 days after Hypr infection. No IFN-β gene expression was detected in infected mice (data not shown). On day two after infection, only gene expression of ISG15 was significantly induced by the virus (Online Resource 1A). The administration of Iristatin did not cause significant changes; however, the non-significant decline in gene expression was seen in 3 out of 4 genes (Online Resource 1A, C, E). Five days after infection, the gene expression of ISG15, CXCL-10, and OASL2 was significantly induced by virus (Fig. 2a, b, Online Resource 1D). The presence of Iristatin resulted in a significant decline of ISG15 (Fig. 2a). The other genes were slightly downregulated (Fig. 2b, Online Resource 1D, F); the CXCL-10 decrease caused by Iristatin was close to statistical significance (*p* = 0.0645). Our results demonstrate a positive correlation between a decline of viral RNA in skin of TBEV-infected mice and the decline of IFN-stimulated gene induction in the presence of Iristatin.

To shed light on the changes in skin that could potentially lead to a decrease of virus load in the presence of Iristatin, we evaluated the presence of monocytes and gamma delta T cells in the skin of TBEV-infected mice 5 dpi. We hypothesized that due to the immunomodulatory effect of Iristatin the altered recruitment of monocytes (virus permissive cells) and/or gamma delta T cells (innate lymphocytes capable to alter cytokine environment in the skin) could be responsible for a decline of virus amount in the skin. The gene expressions of monocyte marker CD115 and gamma delta T cell marker TCRγ were measured; however, no changes were revealed between non-infected and TBEV-infected or Iristatin-treated groups (Fig. 2c, d). The results suggest that Iristatin does not alter the amounts of monocytes or gamma delta T cells in the skin of TBEV-infected mice. Thus, another mechanism of Iristatin-induced viral RNA decline plays a role.

### Viral multiplication is not influenced by Iristatin in mouse macrophages or dendritic cells in vitro

Next, we investigated if Iristatin has a direct inhibitory effect on TBEV replication at the cellular level in vitro. We chose mouse bone marrow macrophages (BMM) and dendritic cells (DC) as they are susceptible to TBEV infection and in addition they play an important role in TBEV pathogenesis and dissemination. Macrophages and DC were infected with Hypr strain at MOI 5 and virus load was determined 24, 48, and 72 h post-infection (hpi). As shown in Fig. 3, no significant changes in viral RNA were observed in the infected macrophages or dendritic cells with or without Iristatin (Fig. 3a, d). The amounts of infectious virus, determined by plaque titration, were also comparable in the presence or absence of Iristatin (Online Resource 2). The gene expression of ISG15 and CXCL-10 was induced upon virus infection in both types of cells; however, it was not affected by Iristatin (Fig. 3b, c, e, f). Interestingly, we consistently observed a tendency of Iristatin to negatively influence the TBEV replication and CXCL-10 gene expression; effects were more pronounced in macrophages than in dendritic cells. Overall, the results suggest that Iristatin does not have a direct effect on the TBEV multiplication in vitro.

### Iristatin inhibits the Erk1/2 activation in TBEV-infected dendritic cells and exerts the anti-apoptotic effect

Signal transduction is fundamental to the understanding of mechanism of action, so we decided to investigate whether signaling pathways activation will be affected by Iristatin. Since Toll-like receptor (TLR) 7 is involved in the recognition of TBEV (Etna et al. 2021), we first performed the screening of intracellular signaling pathways activated by TLR7 ligand imiquimod (IQ). The signaling pathway activation was determined in dendritic cells 3 h upon IQ addition using the PathScan intracellular signaling array kit (only selected kinases are presented in Fig. 4a). Results showed that IQ activated several signaling pathways, including kinases Erk1/2 (extracellular signal-regulated kinases 1 a 2), Akt, Bad, and GSK-3β. The Iristatin presence caused the inhibition of Erk1/2 phosphorylation and enhancement of the phosphorylation of anti-apoptotic Akt and the kinases which are downstream of Akt, namely Bad, mTOR, PRAS40, and GSK-3β. To follow up this observation, we performed the analysis of Erk1/2 and Akt phosphorylation in Hypr-infected DC by immunoblotting. DC were infected by Hypr in the presence or absence of Iristatin and cells were subjected for analysis 1, 2, and 3 h afterwards. The phosphorylation of Akt was gradually increased upon the addition of the virus but the effect of Iristatin was not consistent (data not shown). In the case of Erk1/2 activation, we observed an increase of Erk phosphorylation which was absent in the presence of Iristatin (Fig. 4b). Thus, the results suggest that Iristatin negatively affects the activation of Erk1/2 in virus-infected cells.

Finally, as Iristatin inhibits cathepsin L (Kotal et al. 2019), the lysosomal protease involved in the control of apoptosis, we wondered whether Iristatin would influence the apoptotic process. We measured the active caspase-3, an executive caspase and a marker of apoptotic cells, in the TBEV-infected dendritic cells in the presence or absence of Iristatin 24 and 48 h post-infection. As shown in Fig. 4c, coincubation of DC with the virus led to a minor but statistically significant decrease in the percentage of active caspase-3 positive cells 24 h post-infection. More importantly, Iristatin significantly decreased the percentage of apoptotic cells at both tested intervals independently of virus infection, pointing to the potential of this cystatin to prevent apoptosis.

## Discussion

Cystatins found in arthropods are associated with blood acquisition, digestion, and the modulation of host immune responses and tick immunity (Chmelar et al. 2017; Francischetti et al. 2009; Kotal et al. 2019). In this work, we uncovered a novel role of tick cystatin in relation to TBEV. We have found that the presence of Iristatin, the salivary cystatin of* I. ricinus* tick, led to a decline of viral RNA in the skin of infected mouse. Infected cells upon recognition of viral RNA produce IFN, which exerts its anti-viral effects through induction of numerous IFN-stimulated genes. In agreement with reduced viral RNA, gene induction of chosen IFN-stimulated genes was lowered in skin of infected mice by Iristatin treatment. The effect of Iristatin on TBEV replication in macrophages and dendritic cells in in vitro condition, measured by viral RNA and viral titer, was not observed.

If we presume that viral RNA level correlates with the amount of infectious viral particles, the inhibitory effect we observed by Iristatin in the skin may impact both the host and the tick. Ticks often feed on the same host, and TBEV can be transmitted from a TBEV-infected tick to a non-infected tick by so-called cofeeding with no detectable viremia (Nuttall 2019). This horizontal way of virus transmission is quite common and the reduced load of virus in the skin may negatively affect virus spreading in nature. The importance of reduced virus load for host itself seems obvious if less virus means higher chance for host immune system to eliminate viral infection. In the case of flaviviruses, the initial dose of the virus is not so crucial for pathogenesis (Lennette 1944; Porcelli et al. 2023). Indeed, the administration of Iristatin induced differences at the level of viral RNA in the skin but did not affect the virus load in the brain.

Several studies have described the anti-viral role of vertebrate cystatin (Shah and Bano 2009), and among arthropod cystatins, Aacystatin from *Aedes aegypti* was shown to have an anti-viral effect in mosquitos (Sim et al. 2012). The silencing of the Aacystatin gene led to increased DENV titers and this effect was attributed partially to the modulation of apoptosis (Oliveira et al. 2020b, a). Among tick cystatins, only cystatin Sialostatin L2 from *I. scapularis* was investigated in relation to the TBEV infection. Interestingly, this cystatin potentiated TBEV replication in dendritic cells in vitro and interfered with IFN action (Lieskovska et al. 2015b). Therefore, it earned the mark as a SAT factor, though no in vivo experiments were performed. In contrast to Sialostatin L2, we found that Iristatin does not influence TBEV replication in dendritic cells, so apparently, Iristatin and Sialostatin L2 work in a different manner, possibly due to differences in their substrate specificity (Kotsyfakis et al. 2007). The different modes of action of these two tick cystatins were also observed when investigating the anti-inflammatory effect in a psoriasis-like inflammation model (Wu et al. 2024).

The interaction between vectors and their hosts is very complex and may affect vector-borne pathogens in the host. The modulation of host haemostatic and immune reactions, thanks to molecules present in tick saliva, creates in many cases a favorable microenvironment for tick transmitted pathogens (Nuttall 2019). This was shown for numerous tick-borne pathogens including TBEV, Powassan virus, Thogoto virus, *Borrelia burgdorferi* sensu lato, and *Francisella tularensis* (Hermance and Thangamani 2015; Kazimirova et al. 2017; Nuttall 2019; Simo et al. 2017). Though the mechanism of this supporting effect is not clear, it is believed that in general, an immunosuppression and immunomodulation is involved. Nevertheless, in the case of arboviral infection, it has been shown that the inflammatory response caused by the bite of the mosquito *Aedes aegypti* is needed for the higher load of Semliki Forest virus and Bunyamwera virus (Pingen et al. 2016). Thus, a weaker inflammatory reaction of host may be disadvantageous for virus replication and virus dissemination within the host. We hypothesized that due to the previously reported immunomodulatory and anti-inflammatory features of Iristatin, the recruitment of virus-permissive monocytes to the site of virus inoculation could be negatively affected and consequently could cause a decline in virus load in the skin. Of note, the percentage of innate immune cells including macrophages was decreased by Iristatin in skin lesions and secondary lymphoid organs in mannan-induced inflammation (Kotsyfakis et al. 2007). However, as we found neither virus nor Iristatin cause changes in the amounts of monocytes in the skin of TBEV-infected mice so the mechanism of negative effect of Iristatin remained to be clarified.

The effect of Iristatin on viral replication in vitro was tested as well. The early phase of TBEV infection takes place in cutaneous tissues, where several types of cells including monocytes and Langerhans cells support virus replication (Hermance et al. 2016; Labuda et al. 1996). Therefore, mouse macrophages and dendritic cells were chosen for in vitro analysis of Iristatin effects. However, no changes at the level of viral RNA, the viral titer, or gene expression of IFN-stimulated genes were observed in these cells upon Iristatin treatment. In addition, the preliminary results from testing the murine keratinocytes, the most abundant cells of the epidermis, showed that there is no effect of Iristatin on TBEV replication (data not shown). We think that the lack of Iristatin effect on virus multiplication in vitro implies that Iristatin likely does not interfere with virus replication directly but rather modulates processes important for virus multiplication at the organismal level.

One of the cellular processes which was found to be affected by Iristatin was apoptosis. The percentage of active caspase-3 positive cells out of dendritic cells was substantially decreased by Iristatin in both TBEV-infected and non-infected cells, indicating that the anti-apoptotic effect of Iristatin is independent of virus infection. Interestingly, in in vitro condition, Iristatin negatively influenced the activation of one of mitogen activated kinases Erk1/2, the kinase, which is involved in many cellular processes including proliferation, differentiation, motility, and survival (Cargnello and Roux 2011). The relevance of Erk1/2 inhibition to the TBEV replication in vivo was not determined. However, it has been shown that MEK/ERK inhibitors AZD6244 and UO126 impair the replication of several flaviviruses (Albarnaz et al. 2014; de Oliveira et al. 2020b, a). The inhibition of Erk1/2 signaling by other *I. scapularis* cystatins, Sialostatin L and Sialostatin L2, was also observed in dendritic cells stimulated by TLR ligands or *B. burgdorferi* (Lieskovska et al. 2015a). Thus, the negative effect on Erk1/2 pathway could be a common mode of action of these tick cystatins. Nevertheless, the cellular processes that are altered by Iristatin and important for TBEV replication remain elusive.

Finally, we evaluated the role of Iristatin in the TBEV infection of murine host; however, we do not know what impact Iristatin would have on TBEV infection in ticks. There is no information about Iristatin gene expression in TBEV-infected *I. ricinus* ticks though the study by Hart et al. revealed that one cystatin is downregulated in Hypr-infected *I. ricinus* (Hart et al. 2020) suggesting the possibility of a reciprocal interplay between cystatin and TBEV in ticks. This raises interesting questions that are the aims of our future studies.

## Conclusions

We have discovered that tick salivary cystatin Iristatin acts as a negative regulator of TBEV infection since it can restrict the amount of viral RNA in the host skin. Assuming that the viral RNA correlates with the amounts of infectious viral particles, Iristatin may have an effect on virus transmission among ticks during cofeeding. Importantly, since Iristatin does not behave as a SAT factor, it should not be considered as a part of an anti-tick vaccine for protection against tick-borne pathogens.

## Supplementary Information

Below is the link to the electronic supplementary material.

Supplementary file1 (PDF 157 KB)

## Data Availability

The datasets generated during and/or analysed during the current study are available from the corresponding author on reasonable request.

## Abbreviations

ANOVA: Analysis of variance
BMM: Bone marrow macrophages
DC: Dendritic cell
DENV: Dengue virus
DPI: Days post-infection
ER: Endoplasmic reticulum
GM-CSF: Granulocyte-macrophage colony-stimulating factor
HPI: Hours post-infection
ID: Intradermal
IFN: Interferon
ISG: Interferon-stimulated gene
IQ: Imiquimod
JEV: Japanese encephalitis virus
LCCM: L929 cell conditioned medium
MOI: Multiplicity of infection
PBS: Phosphate-buffered saline
POWV: Powassan virus
SGE: Salivary gland extract
TBEV: Tick-borne encephalitis virus
TLR: Toll-like receptor
WNV: West Nile virus
ZIKV: Zika virus
