# Supplementary material for: Tick salivary cystatin Iristatin limits the virus replication in skin of tick-borne encephalitis virus–infected mice
Source: Parasitol Res. 2025 Jan 17;124(1):8. doi: 10.1007/s00436-024-08441-5 (PMC11739226; doi:10.1007/s00436-024-08441-5)
Supplement: Supplementary file 1 — Supplementary file1 (PDF 157 KB) [file 436_2024_8441_MOESM1_ESM.pdf]

Parasitology Research

Tick salivary cystatin Iristatin limits the virus replication in skin of tick-borne encephalitis virus infected mice

Helena Langhansová [1], Zuzana Beránková [1], Ritesh Khanna [1], Jan Kotál [1], Michail Kotsyfakis [3], Martin Palus[2], Jaroslava Lieskovská [1, 2] *

[1] Department of Medical Biology, Faculty of Science, University of South Bohemia, Branišovská 1760, CZ-37005 České Budějovice, Czech Republic

[2] Institute of Parasitology, Biology Centre of Czech Academy of Sciences, Branišovská 1160/31, CZ-37005 České Budějovice, Czech Republic

[3] Institute of Molecular Biology and Biotechnology, Foundation for Research and Technology-Hellas, N. Plastira 100, 70013, Heraklion, Crete, Greece

*** Correspondence: jlieskovska@prf.jcu.cz**

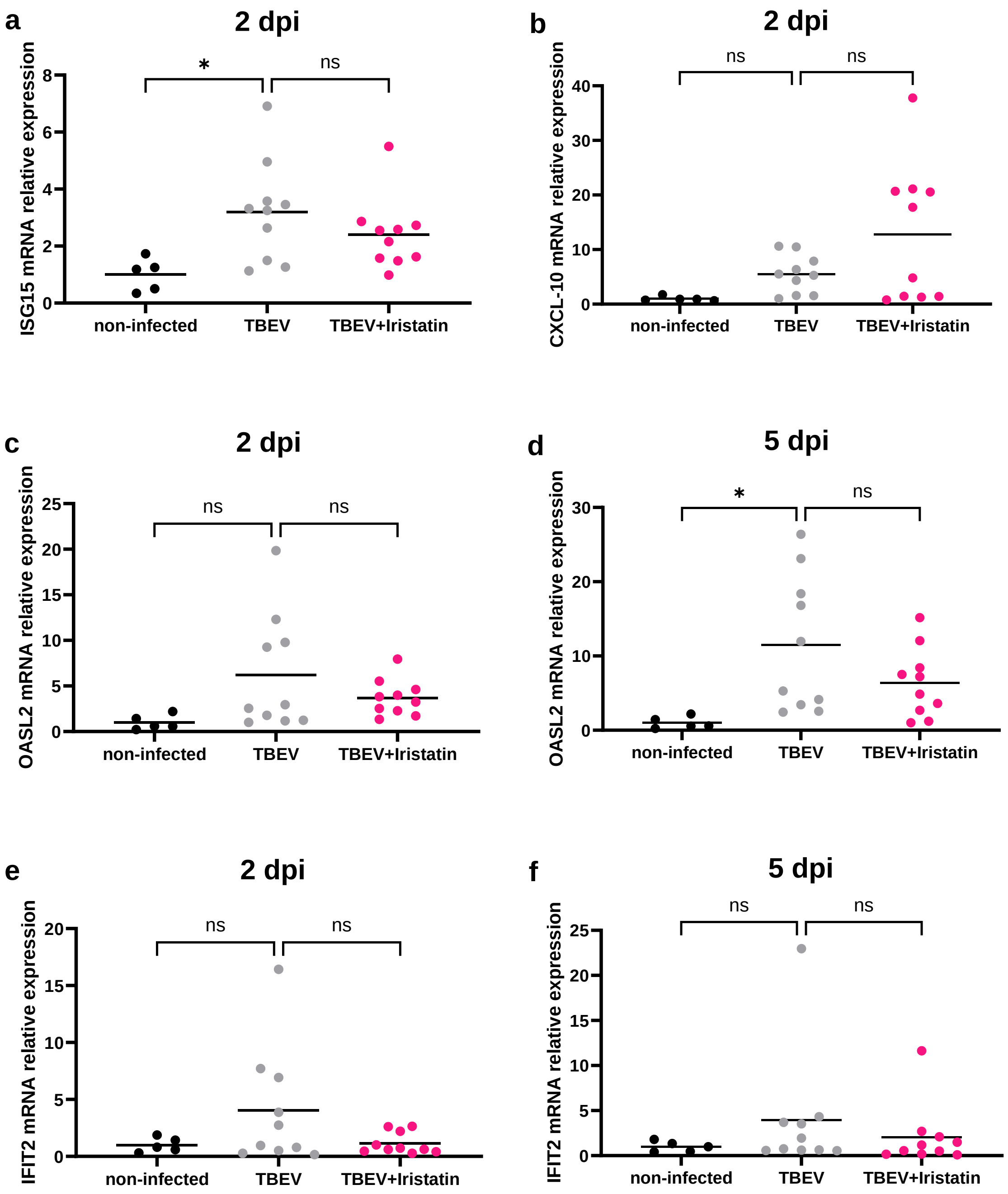

Online Resource 1 The effect of Iristatin on the expression of interferon-responsive genes in the skin of TBEV-infected mice. Mice were i.d. infected with Hypr +/- Iristatin and the gene expressions of ISG15 (**a**), CXCL-10 (**b**), OASL2 (**c, d**), and IFIT2 (**e, f**) were evaluated on depicted days (2 and/or 5 dpi) in the skin of infected mice. mRNA expression was normalized to the Actb mRNA level and non-infected mice. * $p \leq 0.05$; ns = not significant

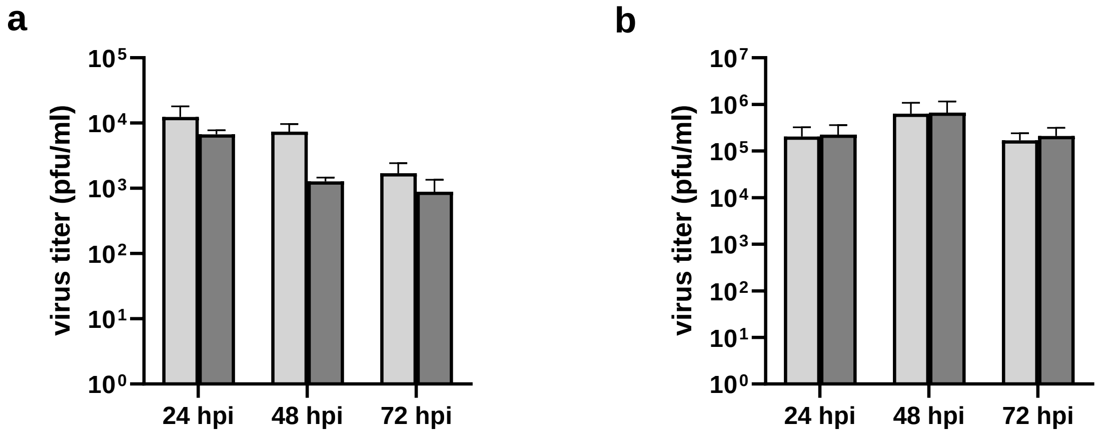

Online Resource 2 The effect of Iristatin on virus multiplication in primary bone marrow macrophages (BMM; **a**) and dendritic cells (DC; **b**). BMM and DC were infected with Hypr (MOI 5) and incubated for 24, 48, and 72 h in the presence or absence of Iristatin (6 µM). Viral titres were determined by plaque assay on indicated hours post infection. The mean of three independent experiments (+ SEM) is shown in the graphs. Differences between groups were not statistically significant.
